# Discovery of novel alkaline-tolerant xylanases from fecal microbiota of dairy cows

**DOI:** 10.1186/s13068-023-02435-8

**Published:** 2023-11-27

**Authors:** Xiaoling Zhang, Qin Miao, Bingling Tang, Ivan Mijakovic, Xiao-Jun Ji, Lingbo Qu, Yongjun Wei

**Affiliations:** 1 School of Pharmaceutical Sciences, Laboratory of Synthetic Biology, Zhengzhou University, Zhengzhou, 450001 China; 2 Division of Systems and Synthetic Biology, Department of Biology and Biological Engineering, Chalmers University of Technology, Gothenburg, 41296 Sweden; 3 The Novo Nordisk Foundation Center for Biosustainability, Technical University of Denmark, Kongens Lyngby, 2800 Denmark; 4 State Key Laboratory of Materials-Oriented Chemical Engineering, College of Biotechnology and Pharmaceutical Engineering, Nanjing Tech University, Nanjing, 211816 China

**Keywords:** Xylo-oligosaccharides, Xylanases, Dairy cow fecal microbiota, Metagenomics, Enzyme characterization

## Abstract

Xylo-oligosaccharides (XOS) are considered as a promising type of prebiotics that can be used in foods, feeds, and healthcare products. Xylanases play a key role in the production of XOS from xylan. In this study, we conducted a metagenomic analysis of the fecal microbiota from dairy cows fed with different types of fodders. Despite the diversity in their diets, the main phyla observed in all fecal microbiota were Firmicutes and Bacteroidetes. At the genus level, one group of dairy cows that were fed probiotic fermented herbal mixture-containing fodders displayed decreased abundance of *Methanobrevibacter* and increased growth of beneficial *Akkermansia* bacteria. Additionally, this group exhibited a high microbial richness and diversity. Through our analysis, we obtained a comprehensive dataset comprising over 280,000 carbohydrate-active enzyme genes. Among these, we identified a total of 163 potential xylanase genes and subsequently expressed 34 of them in *Escherichia coli*. Out of the 34 expressed genes, two alkaline xylanases with excellent temperature stability and pH tolerance were obtained. Notably, CDW-xyl-8 exhibited xylanase activity of 96.1 ± 7.5 U/mg protein, with an optimal working temperature of 55 ℃ and optimal pH of 8.0. CDW-xyl-16 displayed an activity of 427.3 ± 9.1 U/mg protein with an optimal pH of 8.5 and an optimal temperature at 40 ℃. Bioinformatic analyses and structural modeling suggest that CDW-xyl-8 belongs to GH10 family xylanase, and CDW-xyl-16 is a GH11 family xylanase. Both enzymes have the ability to hydrolyze beechwood xylan and produce XOS. In conclusion, this metagenomic study provides valuable insights into the fecal microbiota composition of dairy cows fed different fodder types, revealing main microbial groups and demonstrating the abundance of xylanases. Furthermore, the characterization of two novel xylanases highlights their potential application in XOS production.

**Supplementary Information:**

The online version contains supplementary material available at 10.1186/s13068-023-02435-8.

## Introduction

The increased focus on health-consciousness has led to a growing interest in functional ingredients that could provide nutrition effects and promote overall health [1]. Prebiotics, which are defined as substrates that are selectively utilized by host microorganisms, have a healthy benefit [2], and offer great potential as functional food ingredients in various food and feed products [3]. Among the prebiotics, xylo-oligosaccharides (XOS) have emerged as promising prebiotics with versatile applications in foods, feeds, and healthcare products [4]. XOS exhibit various pharmacological activities, including anti-inflammation, antioxidative, antitumor, and antimicrobial effects [5, 6]. Xylan can be obtained from diverse biomass, including wheat straw, corn straw, rice straw, corncob, rice husk, sugarcane bagasse, poplar, and Chinese hickory shell [7–10]. These readily available and cost-effective raw materials provide abundant resources for XOS production, significantly enhancing the market competitiveness of XOS.

XOS can be produced through chemical degradation method, physical degradation method, and enzymatic degradation method [5, 6, 9–11]. Among these methods, enzymatic degradation is preferred as an ideal method for XOS production, due to its low environmental impact and a simple reaction process that does not require specialized equipments. Moreover, enzymatic degradation has high specificity and minimal production of undesirable byproducts. The enzymatic production of XOS from xylan involves various enzymes, including endo-1,4-β-xylanases, β-xylosidase, α-glucosiduronase, α-l-arabinofuranosidase, acetyl xylan esterase, ferulic acid esterase, and ρ-coumaric acid esterase [12]. Usually, the term “xylanase” refers to endo-1,4-β-xylanases, which are the key enzyme to produce XOS from xylan by hydrolyzing the 1,4-β xylose linkages in the xylan backbone, resulting in the generation of different XOS and small amounts of xylose [13]. Xylanases are mainly found in glycoside hydrolase (GH) families 5, 7, 8, 10, 11, and 43 [14, 15]. Among these families, GH10 and GH11 xylanases are often implemented for XOS production, due to their high specificity and efficiency in breaking down xylan substrates [14, 16].

Currently, xylanases have been isolated from various sources such as bacteria, archaea, fungi, and insects. Generally, microbial xylanases are known for their high thermal stability and alkaline tolerance properties [12, 13]. For example, an endo-xylanase derived from *Aspergillus niger* MTCC 9687 with an optimal temperature of 43.5 ℃ and optimal pH of 5.5, was used to hydrolyze alkali-pretreated cauliflower stalk, resulting in the production of xylobiose as the major XOS [17]. The xylanase of *Paecilomyces variotii*, with an optimal temperature of 60 ℃ and optimal pH of 5.0, could hydrolyze beechwood xylan to produce various XOS [18]. A novel thermostable and alkaline-tolerant GH10 xylanase obtained from alkaliphilic *Bacillus* sp. 30Y5 retained more than 90% activity between pH 6.0 and 9.5, and maintained more than 55% activity at temperature ranging from 50 °C to 70 °C [19]. Most xylanases in the BRENDA database exhibit specific xylanase activity below 500 U/mg protein [19]. Industrial processes are normally carried out at high temperature and extreme pH conditions [20], therefore, the discovery of new xylanase with high activity and high stability is still of great significance for XOS industrial applications.

In the past, xylanases have typically been recovered from isolated strains derived from environmental microbiota [19, 21, 22]. However, this approach is limited, because approximately 99% of all microorganisms found in external environments cannot be cultured in the laboratory. Currently, metagenomics and other microbiome methods have proven to be effective in recovering a large number of xylanase genes from various environments [23–25]. Rich sources of xylanases include microbiota from cow rumen, termite hindgut, biogas digester, and pulp and paper wastewater, which are highly efficient at lignocellulose degradation [26–30]. Especially, the digestive tract ecosystem of herbivores contains highly abundant carbohydrate-active enzymes (CAZymes). Metagenomic sequencing has revealed the presence of numerous glycoside hydrolases existed in digestive microbiota of Saudi sheep and buffalo [31, 32]. Thus, the use of metagenomics to identify xylanase genes from herbivore digestive tract microbiota provides an effective approach for the discovery of new xylanases. However, most of the recovered CAZymes have not been characterized, and characterization of these untapped genes is of interest.

This study employed metagenomic analysis to gain a better understanding of the fecal microbiota in dairy cows fed with different types of fodders. A wide range of lignocellulose-degrading enzymes were identified. Notably, two xylanases, namely CDW-xyl-8 and CDW-xyl-16, were thoroughly characterized, and their ability to produce XOS by breaking down beechwood xylan was evaluated. The findings of this study contribute to the identification of novel xylanases from efficient lignocellulose-degrading microbiota.

## Materials and methods

### Metagenomic sequencing of dairy cow fecal samples

Twelve fecal samples, named CDW1-1–CDW4-3, were collected from twelve healthy dairy cow at Taigong town and Tangzhuang town, Weihui City, Henan Province, China. These cows were cultivated at four different dairy farms and were fed with different fodders (Henan Muyi Animal Pharmaceutical Co., Ltd). Consequently, the 12 dairy cow fecal samples were divided into four groups named CDW1–CDW4 (Additional file 1: Table S1). To minimize potential interference from environmental microorganisms, fresh fecal samples were collected using sterile gloves and transferred into sterile tubes. The samples were then placed in an ice-cold water bath, transported to the lab, and stored at – 80 ℃ until further analysis.

DNeasy PowerSoil Pro Kit (47014, QIAGEN, USA) was used to extracted genomic DNA from 0.25 g fecal sample. A total of 12 genomic DNA samples were obtained and utilized for metagenomic sequencing with the Illumina HiSep 2500 sequencing system (DeepBiome Co., Ltd). The raw sequencing data were processed according to previous description. The clean data were assembled and annotated, and used for the prediction of CAZymes. All the clean data were submitted to SRA database with the accession numbers of SRR22073191-SRR22073202.

### Strains, plasmids and reagents

The *Escherichia coli* strains, TOP10 and BL21(DE3), were obtained from Tolo Biotech Co., Ltd. (Anhui, China). The pET-28a (+) vector was purchased from Genecreate Co, Ltd. (Hubei, China). The beechwood xylan was bought from Megazyme (Bray, Ireland). Xylose, xylobiose, 3,5-dinitrosalicylic acid (DNS), and other chemical reagents were purchased from the Aladdin Biochemical Technology Co., Ltd. (Shanghai, China).

### Screening of novel xylanases genes and phylogenetic analysis

The genes encoding CAZymes in metagenomic sequencing data were predicted with dbCAN V6.0 [33]. All GH genes were extracted from the microbiota presented in the twelve fecal samples. A total of 163 full-length genes (Genbank accession numbers from OR237236 to OR237398) belonging to GH10 or GH11 family were identified from the GH dataset. Among these 163 genes, 34 GH10 or GH11 family genes were selected and named CDW-xyl-1 to CDW-xyl-34. This subset included 31 GH10 genes and 3 GH11 genes (Additional file 1: Table S5). Their relationship with known GH10 and GH11 xylanase genes was evaluated. The amino acid sequences of these 34 candidate xylanase genes, along with 14 known GH10 and GH11 xylanase genes were aligned. The aligned sequences were used to build a phylogenetic tree using MEGA 11.

### Cloning and expression of the 34 candidate xylanase genes

The 34 candidate xylanase genes (CDW-xyl-1 to CDW-xyl-34) were selected for cloning. The cloned xylanase genes were individually inserted into the pET-28a (+) expression vector by the One Step Cloning Kit (Vazyme, Nanjing, China). Consequently, 34 recombinant plasmids were individually used to transform the *E. coli* TOP10 strain. After verification, the correct recombinant plasmids were further introduced into *E. coli* BL21(DE3) strain for protein expression.

The *E. coli* BL21(DE3) strains carrying recombinant plasmids were individually inoculated into 100 mL LB medium with 50 µg/mL kanamycin in a 500-mL shake flask. The cultures were cultivated at 37 ℃ and 200 rpm. Once the OD600 value reached 0.6–0.8, IPTG was added to a final concentration of 200 µM to induce xylanase gene expression. After cultivation for 16 h at 20 ℃, the cell cultures were harvested by centrifugation at 12,000 rpm for 10 min. The harvested cell cultures were washed with phosphate buffered saline (PBS) buffer (pH 7.4) for 3 times, and then suspended in 40 mL lysis buffer with 1 mM phenylmethylsulfonyl fluoride (PMSF, protease inhibitor). The cells were disrupted by supersonic waves (Xiaomei, Kunshan, China) on ice at a power of 100 W for 15 min, with a pulse duration of 3 s and an interval of 5 s. Finally, the cell lysates were centrifuged at 12,000 rpm for 20 min, and the supernatants were used for the evaluation of crude xylanase activity.

To determine the crude xylanase activity, a substrate of 1% beechwood xylan was used, and the DNS method was employed. A total of 50 µL diluted cell supernatants and 50 µL 1% beechwood xylan were mixed and incubated at 20 ℃ for 20 min, 40 ℃ for 20 min, and 60 ℃ for another 20 min. Subsequently, 100 µL of DNS reagent was added to each tube to terminate the enzymatic reaction. The mixtures were then incubated at 95 ℃ for 5 min. The crude activity of the 34 expressed xylanases could be determined [30].

### Expression and purification of CDW-xyl-8 and CDW-xyl-16

The BL21 (DE3) strain harboring CDW-xyl-8 or CDW-xyl-16 was cultivated in 1-L shake flask with 200 mL medium, following the same procedure as described earlier for the expression of the 34 candidate xylanase genes. Subsequently, the cell cultures were harvested, disrupted, and centrifuged, yielding the crude enzyme solution. The Ni NTA beads (Smart-Lifesciences, Jiangsu, China) were used for protein purification. The purified CDW-xyl-8 and CDW-xyl-16 proteins were individually concentrated using 10-kDa ultrafiltration tubes (Merck, Millipore, USA). The purity and molecular weight of CDW-xyl-8 and CDW-xyl-16 proteins were confirmed using sodium dodecyl sulfate–polyacrylamide gel electrophoresis (SDS-PAGE), and the protein concentration was determined using the Bradford method (Sangon Biotech, Shanghai, China).

### Effects of temperature and pH on CDW-xyl-8 and CDW-xyl-16 activity

To determine the optimal temperature for CDW-xyl-8 and CDW-xyl-16, the reaction mixtures were incubated at 20 ℃, 25 ℃, 30 ℃, 35 ℃, 40 ℃, 45 ℃, 50 ℃, 55 ℃, 60 ℃, 65 ℃, 70 ℃, and 75 ℃, separately. The 1% beechwood xylan was prepared in 100 mM Tris–HCl buffer at pH 7.4. The 100 µL reaction mixture contained 50 µL 1% beechwood xylan and 50 µL of appropriately diluted CDW-xyl-8 or CDW-xyl-16 solution. After inoculating at each temperature for 10 min, the reaction was terminated by adding 100 µL DNS reagent, followed by incubation at 95 ℃ for 5 min. The OD540 value of each tube was then detected. The temperature with the highest xylanase activity was considered as the optimal temperature for the respective enzyme.

To determine the optimal pH of CDW-xyl-8 and CDW-xyl-16, the reaction temperature was set to the previously determined optimal temperature for each enzyme. Different buffers covering a wide pH range were used, including disodium phosphate–citrate buffer (200 mM, pH 3.0–6.0), Tris–HCl buffer (100 mM, pH 6.0–9.0), and glycine–NaOH buffer (100 mM, pH 9.0–10.0). The reaction system, reaction time, and xylanase activity detection method were the same as the optimal temperature determination experiment.

To evaluate the temperature stability of CDW-xyl-8 and CDW-xyl-16, each enzyme was separately incubated at 20 ℃, 25 ℃, 30 ℃, 35 ℃, 40 ℃, 45 ℃, 50 ℃, 55 ℃, 60 ℃ or 65 ℃ for 2 h at their respective optimum pH condition. The activity of the enzymes after incubation at different temperature were determined. The highest xylanase activity without incubation was defined as 100%, and the reaction at each temperature was measured in triplicate.

For pH stability, CDW-xyl-8 and CDW-xyl-16 were incubated separately in buffers at different pH (pH range from 5.0 to 10.0) for 2 h at their optimal temperature. The xylanase activity at each pH was then measured. The untreated xylanase activity was defined as 100%, and the activity at each pH condition was measured in triplicate.

### Analysis of XOS generated from beechwood xylan by CDW-xyl-8 or CDW-xyl-16

The products from the hydrolysis of beechwood xylan by CDW-xyl-8 or CDW-xyl-16 were detected using a thin-layer chromatography (TLC) assay. In a 200-µL EP tube, 50 µL 1% beechwood xylan was mixed with 50 µL of appropriately diluted xylanase solution, and reacted for 10 min at the optimal temperature and pH of the enzyme. The reaction was terminated by incubating the tube in a water bath at 100 ℃ for 5 min, followed by centrifuged at 12,000 *g* for 10 min. The resulting enzymatic hydrolysates were analyzed using TLC, with untreated beechwood xylan and inactivated xylanase reacted with beechwood xylan samples serving as negative controls. Xylose and xylobiose standards were used as markers. Silica gel GF254 plates (Qingdao Ocean Chemical Co. Ltd., Qingdao, China) were used as a stationary phase, and the mobile phase was chloroform:acetic acid:water (6:7:1, by volume). The prepared samples (3 µL) were spotted onto the TLC plate, which was then developed until the solvent migrated to approximately 1 cm below the top of the plate. Subsequently, the plate was dried at room temperature. The developed TLC plate was visualized by spraying the color developing agent (diphenylamine/aniline/phosphoric acid/acetone) uniformly, and then heating it in an oven at 100 ℃ for 10 min until brown spots appeared.

### Bioinformatic analyses of CDW-xyl-8 and CDW-xyl-16

The SignalP-6.0 server was used to predict the signal peptide of CDW-xyl-8 and CDW-xyl-16. The SWISS-MODEL server and AlphaFold2 were employed to predict the 3D structure of CDW-xyl-8 and CDW-xyl-16. The resulting modeling structure was visualized and aligned with other known enzyme structures using the PyMOL molecular visualization system. All software parameters are set to their default values.

## Results and discussion

### Metagenomic sequencing of fecal microbiota from dairy cows

A total of 127.4 Gbp clean data were obtained, and metagenomic read assembly resulted in 31,808–232,970 contigs (≥ 1000 bp) from the 12 fecal samples (Additional file 1: Table S2). The number of open reading frames (ORF) in each sample ranged from 1,424,459 to 5,312,317 and the average ORF length ranged from 296.83 to 391.62 bp, showing most ORFs were not in full-length (Additional file 1: Table S3). Among these fecal microbiota, the sequence data of CDW4-2 are insufficient, resulting in low sequence coverage.

Metagenomic analysis showed that there were more than 100 phyla in each dairy cow fecal microbiota, dominated by Firmicutes, Bacteroidota, and another 5 phyla (Additional file 1: Fig. S1A). Firmicutes and Bacteroidota accounted for an average of 70.11% in CDW1 group, 68.96% in CDW2 group, 71.94% in CDW3 group, and 68.15% in CDW4 group. Other dominant phyla include Proteobacteria, Verrucomicrobiota, Actinobacteria, Spirochaetota, and Euryarchaeota. The microbial distribution was similar to the fecal microbial profiles of cattle fed with coconut coir, a high-fiber diet, or a normal diet [34–36]. At the genus level, *Prevotella*, *Bacteroides*, *Ruminococcus*_E, *Akkermansia*, *Methanobrevibacter*_A, and *Clostridium* were the dominant genera (Additional file 1: Fig. S1B). The abundance of *Methanobrevibacter* in CDW1 group was significantly lower compared to the other three groups (Additional file 1: Figs. S1B, S2A, S2B), suggesting that probiotic fermented herbal mixture in CDW1 might inhibit the growth of *Methanobrevibacter*, leading to a reduction in methane gas emissions. On the other hand, the richness of *Akkermansia* in CDW1 group was significantly higher than that in the other three groups (Additional file 1: Figs. S1B, S2C). *Akkermansia* plays a critical role in maintaining homeostatic immunity and positively regulating host metabolism [37, 38], hinting that the addition of probiotic fermented herbal mixture added in the CDW-1 group promoted the growth of beneficial *Akkermansia* bacteria. Overall, the probiotic fermented herbal mixture in dairy cow fodder has the potential to inhibit the growth of pathogenic bacteria while promoting the growth of beneficial bacteria.

The fecal microbiota in the four groups fed with different diets exhibited significant changes. The α-diversity analysis showed that the microbial composition in CDW1 had a significantly higher richness and diversity compared to the other groups (Additional file 1: Table S4). The β-diversity analysis indicated that the three samples in CDW1 group were similar, while samples from the CDW2, CDW3, and CDW4 groups showed differences (Additional file 1: Fig. S3). The CDW1 group, which was supplemented with probiotic fermented herbal mixture in the fodder, displayed the highest microbial species richness and diversity, with a stable microbial composition across the three samples. This may be explained by the capacity of the probiotic fermented herbal mixture to promote the growth of probiotics in dairy cow, thereby maintaining the homeostasis of rumen environment [39, 40]. However, further verification in a large dairy cow cohort is necessary to confirm the effects of reducing methane release and promoting microbial growth.

### Construction of a dataset of carbohydrate-active enzyme (CAZyme) genes

Metabolic analysis based on functional genes revealed that carbohydrate metabolic genes were the most abundant category in the fecal samples (Additional file 1: Fig. S4A). Specifically, the CDW1 group had an average of 128,737 carbohydrate metabolic genes, the CDW2 group had an average of 116,167 genes, the CDW3 group had an average of 100,249 genes, and the CDW4 group had an average of 120,474 genes (Additional file 1: Fig. S4B). The abundance of these carbohydrate metabolic genes suggested that the dairy cow fecal microbiota may possess a strong ability to degrade lignocellulose. Since dairy cow fodders often contained a high amount of lignocellulose, it is likely that the fecal microbiota is a rich reservoir of xylanases and other lignocellulose-degrading enzymes. Among the four groups, the CDW1 group contained more carbohydrate metabolic genes compared to the other three groups (Additional file 1: Fig. S4B), suggesting that the CDW1 group dairy cows exhibited high lignocellulose degradation ability, possibly due to the inclusion of fermented herbs in their fodders.

The online tool dbCAN2 analysis results confirmed the presence of various CAZymes (>280,000 genes) in the dairy cow fecal microbiota, including glycoside hydrolases (GHs), glycosyl transferases (GTs), polysaccharide lyases (PLs), carbohydrate esterases (CEs), auxiliary activities (AA), and carbohydrate binding modules (CBMs) [41]. The average number of total CAZyme genes in fecal samples of CDW1 group was 78,142, which is significantly higher than that in the other three groups (69,729 CAZyme genes for CDW2 group, 63,912 CAZyme genes for CDW3 group, and 70,981 CAZyme genes for CDW4 group) (Additional file 1: Fig. S5A). These CAZyme genes provide a valuable resource for the discovery of diverse functional CAZymes. Compared to the other three dairy cow groups, the inclusion of probiotic fermented herbs in the diet of the CDW1 group appears to promote the accumulation of diverse lignocellulose degradation genes in the fecal microbiota of dairy cows.

### Selection of candidate xylanase genes and determination of crude xylanase activity

Most xylanases used in XOS production belong to the GH10 and GH11 families. In this study, we identified a total of 2225 GH10 genes and 194 GH11 genes. Among these genes, 158 GH10 genes and 5 GH11 genes were predicted to be full-length. The number of GH10 and GH11 genes in CDW1 group was significantly higher than that in the other three groups (Additional file 1: Fig. S5B). Besides, the number of GH10 genes was significantly higher than that of GH11 genes (Additional file 1: Fig. S5B). Compared with GH11 family, GH10 family xylanases are more active towards the xylan backbone and more thermostable during biomass degradation. Therefore, the presence of more GH10 xylanases in the fecal microbiota of dairy cows is beneficial for efficient lignocellulose and fodder digestion.

**Fig. 1:**
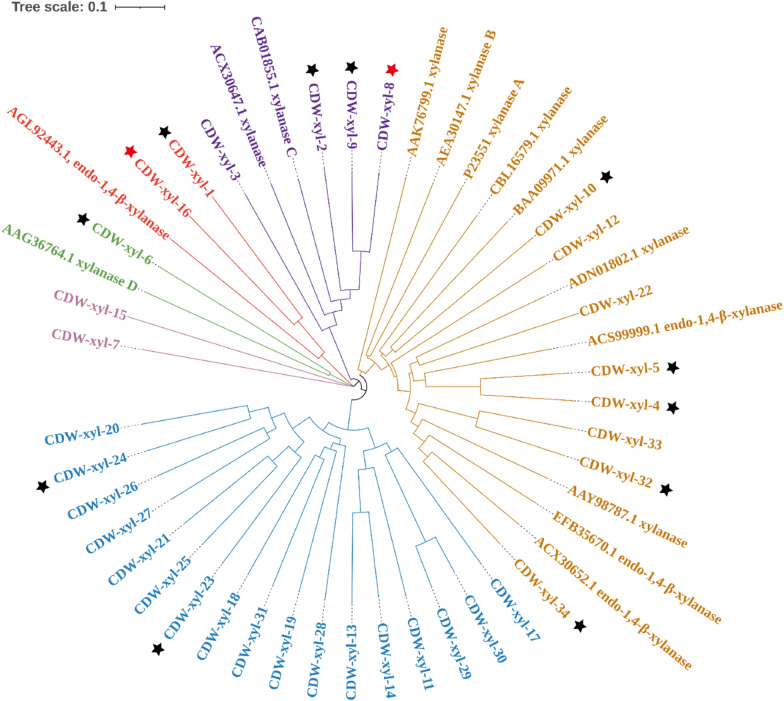
The phylogenetic tree of the 34 candidate xylanase genes and 14 known active xylanases. A known endo-1,4-β-xylanase (GenBank ID: AGL92443.1) was from GH11 family, and the other 13 known xylanases were from GH10 family. The 13 expressed candidate xylanases displayed xylanase activity were marked with stars. CDW-xyl-8 and CDW-xyl-16 were marked with red stars

The 158 GH10 genes and 5 GH11 genes were assigned into several clusters based on phylogenetic analysis (Additional file 1: Fig. S6). From these clusters, a total of 34 candidate xylanase genes were selected, including 31 GH10 genes and 3 GH11 genes (CDW-xyl-1, CDW-xyl-15, and CDW-xyl-16) (Additional file 1: Fig. S6, Table S5). The protein sequences of the 34 xylanases were different from the 14 known active xylanases (Fig. 1), suggesting they had certain novelty. Among them, CDW-xyl-1 and CDW-xyl-16 clustered with a known GH11 active xylanases (GenBank ID: AGL92443.1) from alkaliphilic *Bacillus* sp. SN5. The length of these selected xylanase genes ranged from 1137 to 3912 bp, and they showed 44.66–99.87% sequence identities with known gene sequences (Additional file 1: Table S6). Some of the recovered xylanase genes shared high sequence identities with known genes derived from other lignocellulose degradation microbiota, suggesting their potential involvement in different lignocellulose degradation systems and their effectiveness in xylan degradation. However, these selected genes had not been characterized before. In many large metagenomic datasets, predicted genes often remain uncharacterized, resulting in a significant amount of untapped data. Therefore, it is crucial to further characterize these xylanase genes to harness their potential as efficient xylanases and exemplify the responsible utilization of metagenomic data in the industry. By conducting detailed characterization studies, we can unlock the full potential of these genes and demonstrate the value of employing metagenomic information in industrial applications.

The crude enzyme activity analysis showed that 13 out of the 34 expressed putative xylanases (38.24%) displayed xylanase activity (Fig. 1; Additional file 1: Fig. S7), indicating that the metagenomic screening of xylanases was effective. Among the 13 active xylanases, CDW-xyl-8 and CDW-xyl-16 exhibited the highest xylanase activity (Additional file 1: Fig. S7). CDW-xyl-8 had 66.61% sequence identity with an endo-1,4-β-xylanase of GH10 family derived from *Bacteroidales* bacterium, while CDW-xyl-16 had 98.74% sequence identity with a GH11 genes derived from *Fibrobacteraceae* bacterium (Additional file 1: Table S6). As CDW-xyl-8 and CDW-xyl-16 exhibited relatively high crude enzyme activity, they were selected for further purification and characterization.

### Purification and characterization of CDW-xyl-8 and CDW-xyl-16

**Fig. 2:**
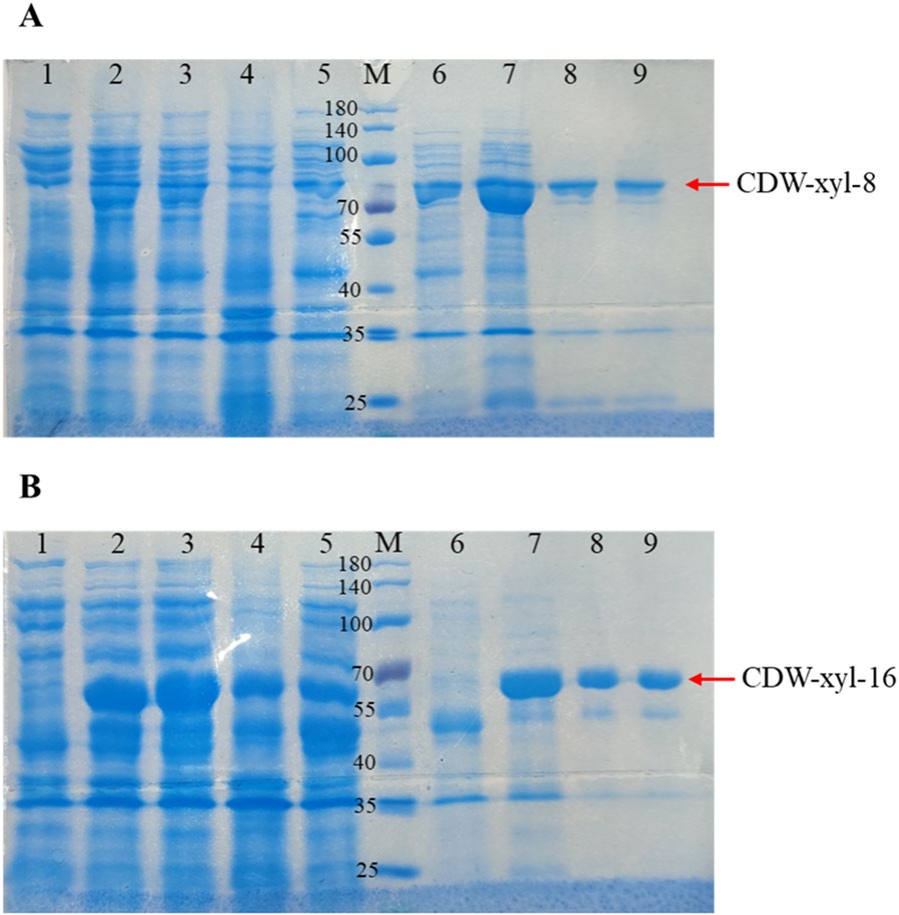
The protein purification of CDW-xyl-8 and CDW-xyl-16. **A** Lane 1, protein supernatant of *E. coli* BL21 (DE3) expressed empty plasmid as a negative control; Lane 2, total protein mixture of *E. coli* BL21 (DE3) expressed CDW-xyl-8; Lane 3, supernatant protein mixture of *E.coli* BL21 (DE3) expressed CDW-xyl-8; Lane 4, precipitated protein mixture of *E. coli* BL21 (DE3) expressed CDW-xyl-8; Lane 5, Ni column flow-through solution after protein absorption; Lane 6, protein washed with 20 mM imidazole buffer; Lane 7, protein washed with 50 mM imidazole buffer; Lane 8, protein washed with 100 mM imidazole buffer; Lane 9, protein washed with 300 mM imidazole buffer; Lane M, molecular mass standards. **B** The same sequence of loading samples for CDW-xyl-16 SDS-PAGE results

**Fig. 3:**
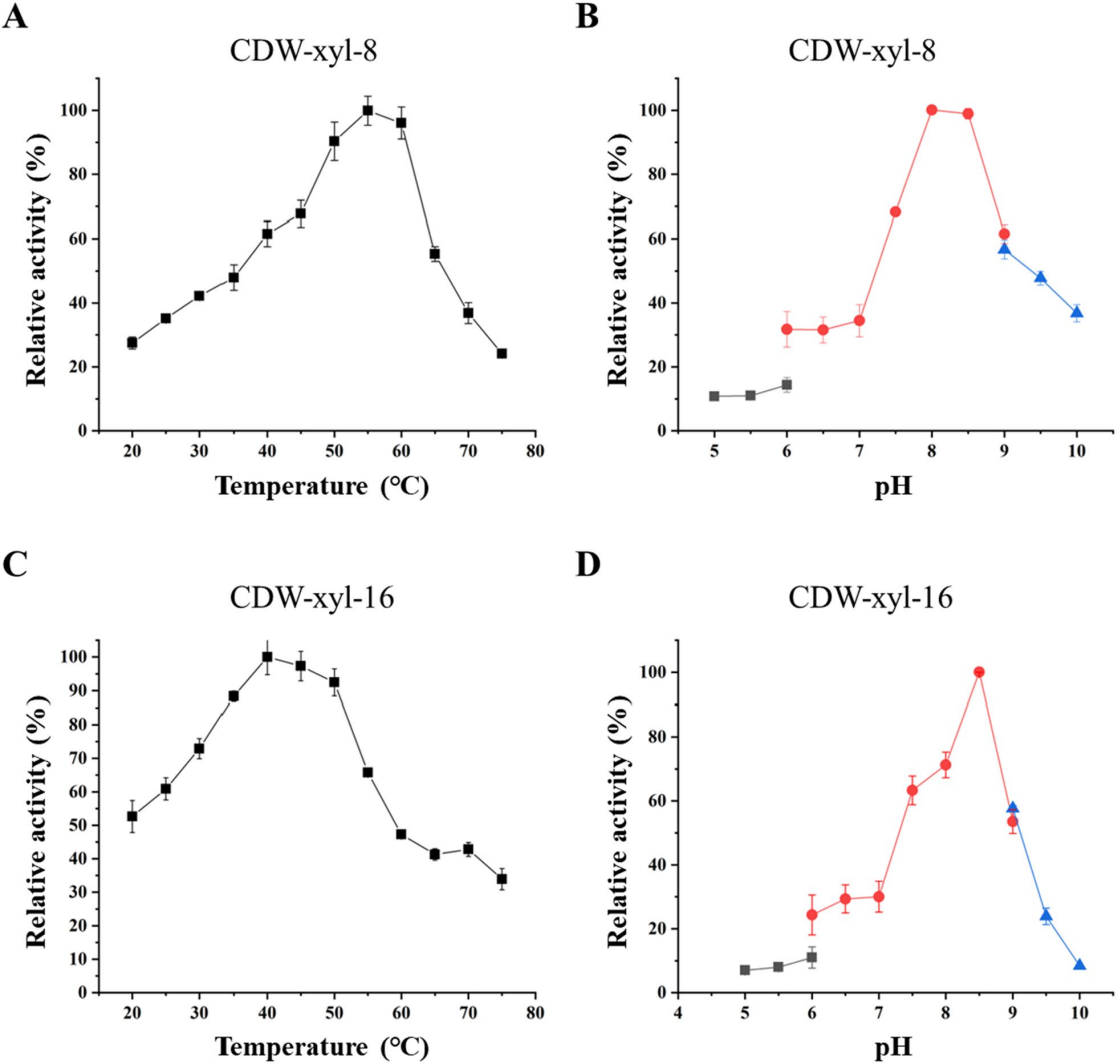
The enzymatic characteristics of CDW-xyl-8 and CDW-xyl-16. **A** The optimal temperature of CDW-xyl-8; **B** The optimal pH of CDW-xyl-8; **C** The optimal temperature of CDW-xyl-16; **D** The optimal pH of CDW-xyl-16. The pH conditions of the dark grey data dots were provided by disodium phosphate–citrate buffer (200 mM, pH 3.0–6.0), the pH conditions of the red data dots were provided by Tris–HCl buffer (100 mM, pH 6.0–9.0), and the pH conditions of the blue data dots were provided by glycine–NaOH buffer (100 mM, pH 9.0–10.0) in **B** and **D**. The values represent the mean values of triplicate experiments, and the error bar indicates the standard deviation

**Fig. 4:**
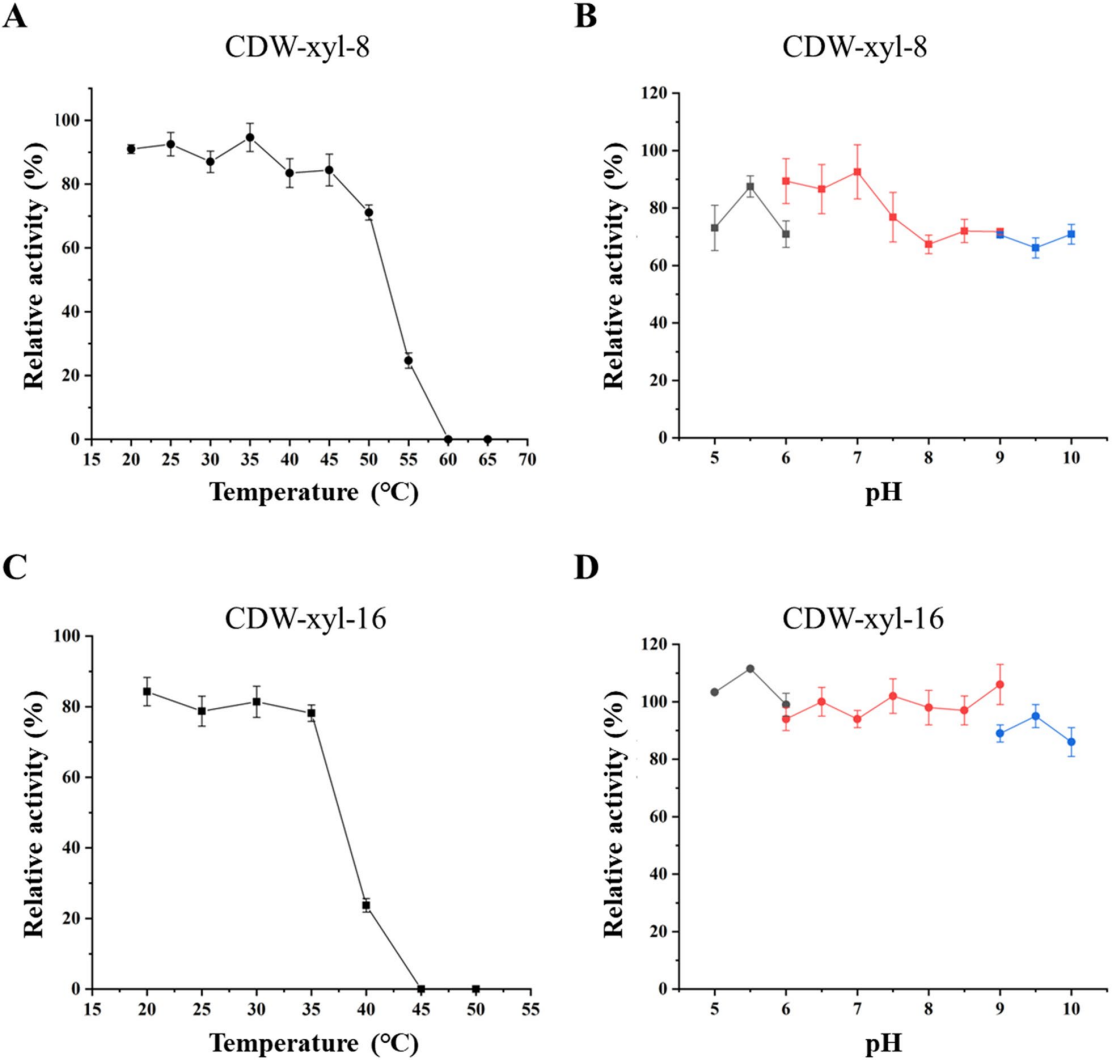
Temperature stability and pH tolerance of CDW-xyl-8 and CDW-xyl-16. **A** Temperature stability of CDW-xyl-8; **B** pH tolerance of CDW-xyl-8; **C** Temperature stability of CDW-xyl-16; **D** pH tolerance of CDW-xyl-16. The buffer used for different pH conditions in **B** and **D** were same as previous descriptions in Fig. 3. The values represent the mean values of triplicate experiments, and the error bar indicates the standard deviation

CDW-xyl-8 and CDW-xyl-16 were expressed in *E. coli*, and the xylanases were successfully purified (Fig. 2). The calculated molecular weights of CDW-xyl-8 and CDW-xyl-16 were 68.5 kDa and 51.9 kDa, respectively, and their purified proteins are similar with their calculated molecular weight values (Fig. 2). The optimal temperature and pH of CDW-xyl-8 were 55 ℃ and pH 8.0, respectively (Fig. 3A, B), while the optimal temperature and pH of CDW-xyl-16 were 40 ℃ and pH 8.5, respectively (Fig. 3C, D). Both xylanases were alkaline tolerant, and the optimal temperature of CDW-xyl-8 was higher than that of CDW-xyl-16. The xylanase activity of CDW-xyl-8 at its optimal condition was 96.1 ± 7.5 U/mg, while the activity of CDW-xyl-16 at its optimal condition was 427.3 ± 9.1 U/mg. CDW-xyl-8 could maintain > 70% xylanase activity after incubated at 20 ℃ to 50 ℃ for 2 h (Fig. 4A). CDW-xyl-16 could maintain > 75% activity after incubated at 20 ℃ to 35 ℃ for 2 h (Fig. 4C). The xylanase activity of CDW-xyl-8 could maintain > 70% activity from pH 6.0 to pH 7.5 after 2 h incubation, and keep > 60% activity from pH 5.0 to pH 10.0 (Fig. 4B). The xylanase activity of CDW-xyl-16 maintained > 80% activity from 5.0 to pH 10.0 after 2 h incubation (Fig. 4D). Although CDW-xyl-8 showed better temperature resistance than CDW-xyl-16, CDW-xyl-16 had better pH tolerance than CDW-xyl-8.

Alkaline pre-treatment is widely regarded as one of the most effective methods for the production of XOS from lignocellulosic biomass [7, 42]. The use of thermo-alkaline xylanase enables direct enzymatic hydrolysis, eliminating the need for additional temperature and pH adjustments, thereby saving costs and time [43]. Compared to the xylanases of PW-xyl9 and PW-xyl37 identified in the pulp and paper wastewater treatment microbiota [44], CDW-xyl-8 and CDW-xyl-16 exhibit superior pH tolerance. Therefore, CDW-xyl-8 and CDW-xyl-16 have promising potential application in prebiotic production, due to their excellent temperature and pH stability. Furthermore, CDW-xyl-8 and CDW-xyl-16 have other potential biotechnological applications, including pulp and paper industry, textile processes, and waste treatments, for these industrial processes often require high temperature and alkaline pH conditions [20, 45].

### Bioinformatic analysis and xylan hydrolytic activity confirmation of CDW-xyl-8 and CDW-xyl-16

**Fig. 5:**
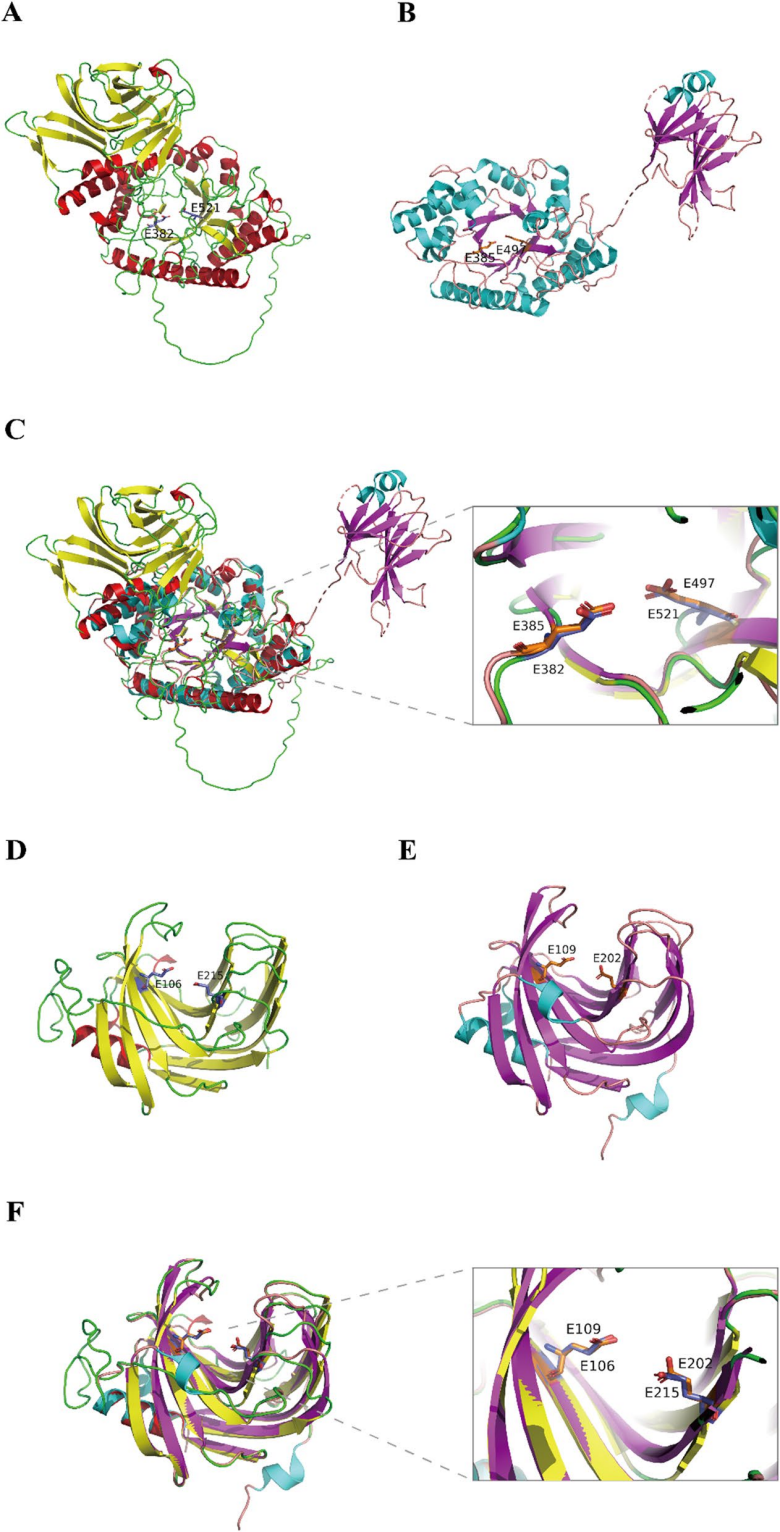
The modeling structures and structure comparisons of CDW-xyl-8 and CDW-xyl-16. **A** The predicted cartoon structure of CDW-xyl-8 by AlphaFold2, and its catalytic residues are E382 and E521. **B** The cartoon structure of *Cj*Xyn10C (PDB ID: 1US3), a known GH10 family xylanase, and its catalytic residues are E385 and E497. **C** Superimposition of CDW-xyl-8 model and *Cj*Xyn10C, they both adopted a typical (*β/α*)_8_ barrel fold of GH10 family xylanase, and the catalytic sites overlapped well. **D** The predicted cartoon structure of CDW-xyl-16 by SWISS-MODEL, and its catalytic residues are E106 and E215. **E** The cartoon structure of XynCDBFV (PDB ID: 3WP4), a known GH11 family xylanase, and its catalytic residues are E109 and E202. **F** Superimposition of CDW-xyl-16 model and XynCDBFV, they both displayed a typical *β*-jelly-roll fold of GH10 family xylanase, and the catalytic sites overlapped well. The *α*-helixes are colored with red, *β*-sheets are colored with yellow, turns and loops are colored with green, and the C atoms of catalytic residues are colored with bule in CDW-xyl-8 and CDW-xyl-16. The *α*-helixes are colored with cyan, *β*-sheets are colored with magenta, turns and loops are colored with salmon, and the C atoms of catalytic residues are colored with orange in *Cj*Xyn10C and XynCDBFV

CDW-xyl-8 has 617 amino acids, and its theoretical pI value is 4.49. Signal 6.0 predicted that CDW-xyl-8 had a lipoprotein signal peptide (Sec/SPII), with a cleavage site between amino acids 23 and 24 (probability was 0.686) (Additional file 1: Fig. S8). Sec/SPII is a secretory signal peptide, suggesting that CDW-xyl-8 is a secretory xylanase. An GH10 xylanase, *Cj*Xyn10C from *Cellvibrio japonicus* (PDB ID: 1US3) (Fig. 5B) [46], was used to predict the structure of CDW-xyl-8 in SWISS-MODEL server. The sequence identity between CDW-xyl-8 and *Cj*Xyn10C is 31.27%. The modeling results showed that CDW-xyl-8 adopted a typical (*β/α*)_8_ barrel fold of GH 10 family xylanase in its core catalytic domain. However, the additional region does not form a proper fold. This could potentially be attributed to the low sequence identity between CDW-xyl-8 and the template structure (1US3) used in SWISS-MODEL modeling.

AlphaFold2 [47] was implemented to predict the structure of CDW-xyl-8, showing that CDW-xyl-8 had a typical (*β/α*)_8_ barrel fold, along with an additional domain (Fig. 5A). The catalytic residues E382 and E521 predicted from sequence alignment of C*j*Xyn10C were found in the corresponding position of CDW-xyl-8 (Fig. 5A), and E382 and E521 overlapped well with the catalytic residues E385 and E497 of C*j*Xyn10C (Fig. 5B, C). The extra domain (Residues 180–350) of CDW-xyl-8 showed 52% sequence identity with a carbohydrate binding domain (CBM) sequence derived from an endo-1,4-beta-xylanase of *Bacteroides gallinaceum*. To gain insights into the newly formed domain of CDW-xyl-8, structure comparison were performed with the CBM15 module of C*j*Xyn10C [46], and a CBM15 module of xylanase Xyn10C derived from *Cellvibrio japonicus* [48]. CDW-xyl-8 displayed a similar *β*-jelly-roll fold structural feature (Fig. 5A–C; Additional file 1: Fig. S9), suggesting that the CBM-like module of CDW-xyl-8 may have substrate-binding function. The CBM-like module is located within the middle sequence of CDW-xyl-8, which is different from the N-terminal CBM15 attached to the catalytic domain of C*j*Xyn10C [46]. The influence of the distinct structural feature on CDW-xyl-8 activity needs further investigation.

CDW-xyl-16 has 478 amino acids, and its theoretical pI value is 4.85. Signal 6.0 predicted that CDW-xyl-16 had no signal peptide. A thermophilic xylanase, XynCDBFV of GH11 family from *Neocallimastix patriciarum* (PDB ID: 3WP4) (Fig. 5E) [49], was used as the template to predict the structure of CDW-xyl-16 in SWISS-MODEL server. The sequence identity between CDW-xyl-16 and XynCDBFV is 42.58%. The GMQE value of CDW-xyl-16 model structure is 0.33, and the QMEANDisCo Global value is 0.68 ± 0.06. CDW-xyl-16 displayed a *β*-jelly-roll fold (Fig. 5D, F), which was the typical structure of GH11 family enzymes. CDW-xyl-16 had a tunnel-like active site cleft formed by the curved inner *β*-sheet, which was similar to XynCDBFV (Fig. 5D, E). The catalytic residues E106 and E215 predicted from sequence alignment of XynCDBFV were found embedded in this region of CDW-xyl-16 (Fig. 5D), and E106 and E215 overlapped well with the catalytic residues E109 and E202 of XynCDBFV (Fig. 5F). Besides, the N-terminal region of CDW-xyl-16 showed high sequence identity with the N-terminal region (NTR) of XynCDBFV and other GH11 family xylanase (Additional file 1: Fig. S10). NTR is unique in the GH11 family, and NTR plays a role in XynCDBFV thermostability [49], which suggested that the NTR of CDW-xyl-16 might be important for its catalytic activity and stable properties.

**Fig. 6:**
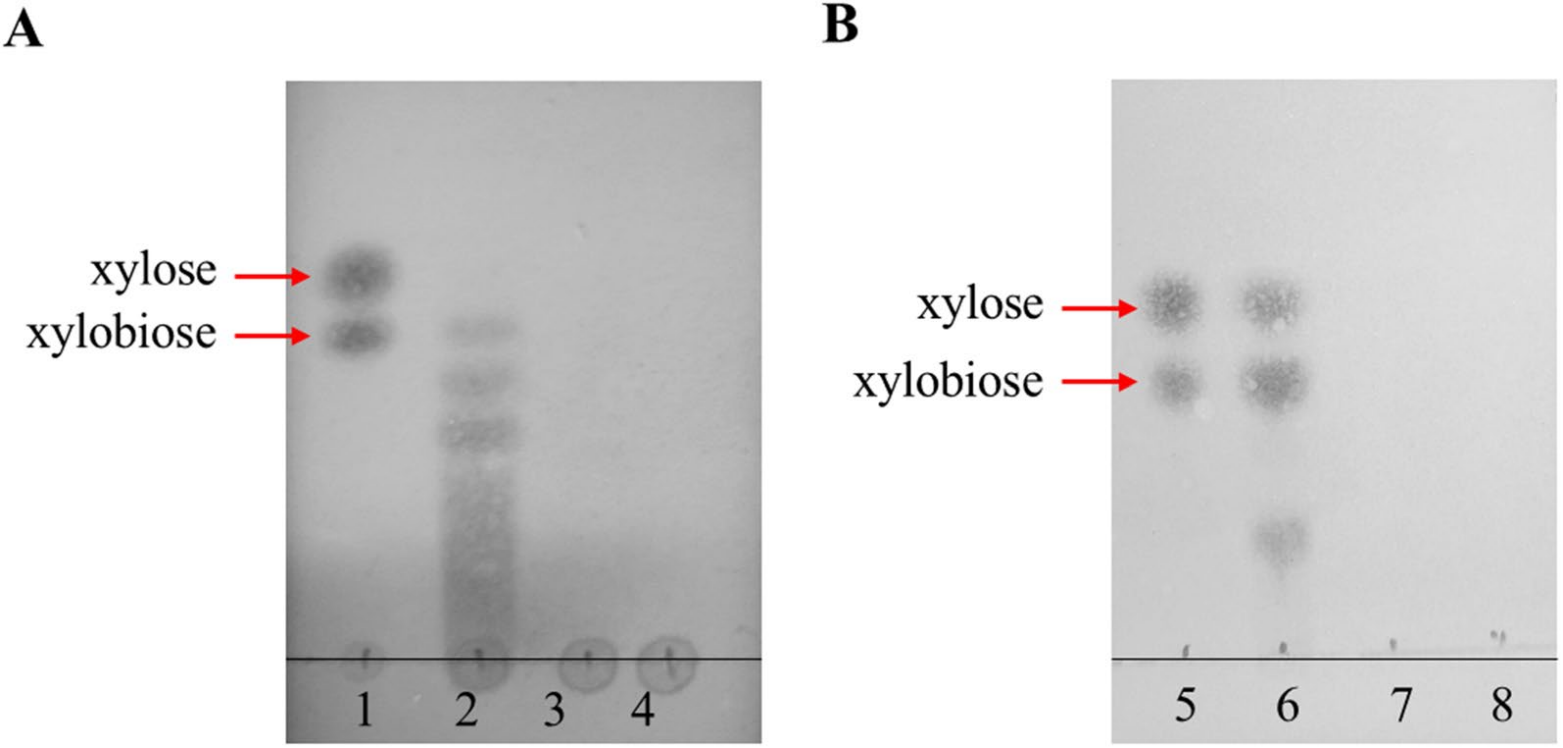
The hydrolysates of beechwood xylan by CDW-xyl-8 or CDW-xyl-16. **A** Line 1: xylose and xylobiose (marker). Line 2: the hydrolysates of beechwood xylan by CDW-xyl-8. Line 3: the hydrolysates of beech xylan by inactivated CDW-xyl-8. Line 4: beechwood xylan (negative control). **B** Line 5: xylose and xylobiose (marker). Line 6: the hydrolysates of beechwood xylan by CDW-xyl-16. Line 7: the hydrolysates of beech xylan by inactivated CDW-xyl-16. Line 8: beechwood xylan (negative control)

The degradation ability of CDW-xyl-8 and CDW-xyl-16 on beechwood xylan were evaluated by TLC. The hydrolysis products of CDW-xyl-8 included xylobiose and a series of other different degrees oligosaccharides without xylose, which could avoid xylose inhibitory effect on xylanase activity [13], showing CDW-xyl-8 has the potential to produce XOS directly in the future (Fig. 6A). The hydrolysis products of CDW-xyl-16 were relatively simple, mainly consisting of xylose, xylobiose, and oligosaccharides (Fig. 6B). Both CDW-xyl-8 and CDW-xyl-16 have the ability to hydrolyze beechwood xylan and generate different types of XOS. The pattern of oligosaccharides obtained is to some extent reported to be GH-family and enzyme dependent [50]. Therefore, the xylanase with different characteristics should be selected according to the actual demand in XOS productions.

## Conclusions

In this study, we investigated the fecal microbiota of 12 dairy cows that were fed with different fodders. Our analysis revealed a rich reservoir of CAZyme genes in the fecal microbiota of the dairy cows. We screened out 163 full-length xylanase genes from GH10 and GH11 families, and selected 34 xylanase genes from various gene clusters for further analyses. Among the 34 expressed xylanases, 13 xylanases showed the ability to hydrolyze beechwood xylan. Further investigation identified two xylanases of CDW-xyl-8 and CDW-xyl-16, which were alkaline xylanase and exhibited excellent stability under different temperature and pH. CDW-xyl-8 and CDW-xyl-16 demonstrated the ability to hydrolyze beechwood xylan into various XOS, which have promising potential for application in XOS production. In summary, our study highlights the ability of the metagenomic strategy in recovering novel xylanases capable of producing XOS and other efficient lignocellulose degradation enzymes, which might pave the way to further advancement in lignocellulose studies.

### Supplementary Information

**Additional file 1: Fig. S1** The microbial compositions in different dairy cow fecal samples. **Fig. S2** Three microbial genera of CDW-1 group were different from other groups. **Fig. S3** The β-diversity of microbiota in different dairy cow fecal samples. **Fig. S4** The annotated gene functions of KEGG pathway. **Fig. S5** The numbers and classification of carbohydrate-active enzyme genes in different sample groups. **Fig. S6** The phylogenetic tree of the predicted 163 genes (158 GH10 genes and 5 GH11 genes). **Fig. S7** The crude enzyme activities of the selected 34 candidate xylanase genes. **Fig. S8** Signal peptide prediction of CDW-xyl-8 by SignalP 6.0 server. **Fig. S9** Local quality estimate of the built model of CDW-xyl-8. **Table S1.** The information of the dairy cows. **Table S2.** Metagenomic data assembly results. **Table S3.** Open reading frame (ORF) data of each sample. **Table S4.** The α-diversity values of microbiota in different dairy cow fecal sample groups. **Table S5.** The xylanase genes number, GH-family classification, gene sequences ID in our dataset, and Genbank accession numbers for the 34 candidate xylanase genes. **Table S6.** Homology analysis of 34 predicted xylanase protein sequences by BLAST in NCBI.

## Data Availability

Data are available on request from the authors.
