# Supplementary material for: Discovery of novel alkaline-tolerant xylanases from fecal microbiota of dairy cows
Source: Biotechnol Biofuels Bioprod. 2023 Nov 27;16:182. doi: 10.1186/s13068-023-02435-8 (PMC10683242; doi:10.1186/s13068-023-02435-8)
Supplement: Supplementary file 1 — Additional file 1: Fig. S1 The microbial compositions in different dairy cow fecal samples. Fig. S2 Three microbial genera of CDW-1 group were different from other groups. Fig. S3 The β-diversity of microbiota in different dairy cow fecal samples. Fig. S4 The annotated gene functions of KEGG pathway. Fig. S5 The numbers and classification of carbohydrate-active enzyme genes in different sample groups. Fig. S6 The phylogenetic tree of the predicted 163 genes (158 GH10 genes and 5 GH11 genes). Fig. S7 The crude enzyme activities of the selected 34 candidate xylanase genes. Fig. S8 Signal peptide prediction of CDW-xyl-8 by SignalP 6.0 server. Fig. S9 Local quality estimate of the built model of CDW-xyl-8. Table S1. The information of the dairy cows. Table S2. Metagenomic data assembly results. Table S3. Open reading frame (ORF) data of each sample. Table S4. The α-diversity values of microbiota in different dairy cow fecal sample groups. Table S5. The xylanase genes number, GH-family classification, gene sequences ID in our dataset, and Genbank accession numbers for the 34 candidate xylanase genes. Table S6. Homology analysis of 34 predicted xylanase protein sequences by BLAST in NCBI. [file 13068_2023_2435_MOESM1_ESM.docx]

Additional file 1

**Discovery of novel alkaline tolerant xylanases from fecal microbiota of dairy cows**

Xiaoling Zhang^a^, Qin Miao^a^, Bingling Tang^a^, Ivan Mijakovic^b,c^, Xiao-Jun Ji^d^, Lingbo Qu^a^, Yongjun Wei^a*^

^a^ School of Pharmaceutical Sciences, Laboratory of Synthetic Biology, Zhengzhou University, Zhengzhou 450001, People's Republic of China

^b^ Division of Systems and Synthetic Biology, Department of Biology and Biological Engineering, Chalmers University of Technology, Gothenburg, Sweden

^c^ Novo Nordisk Foundation Center for Biosustainability, Technical University of Denmark, Lyngby, Denmark

^d^ State Key Laboratory of Materials‐Oriented Chemical Engineering, College of Biotechnology and Pharmaceutical Engineering, Nanjing Tech University, Nanjing, People's Republic of China

^*^ Corresponding author

Yongjun Wei

E-mail: yongjunwei@zzu.edu.cn

**Supplementary figures and tables**

**Fig. S1** The microbial compositions in different dairy cow fecal samples.

**Fig. S2** Three microbial genera of CDW-1 group were different from other groups.

**Fig. S3** The β-diversity of microbiota in different dairy cow fecal samples.

**Fig. S4** The annotated gene functions of KEGG pathway.

**Fig. S5** The numbers and classification of carbohydrate-active enzyme genes in different sample groups.

**Fig. S6** The phylogenetic tree of the predicted 163 genes (158 GH10 genes and 5 GH11 genes).

**Fig. S7** The crude enzyme activities of the selected 34 candidate xylanase genes.

**Fig. S8** Signal peptide prediction of CDW-xyl-8 by SignalP 6.0 server.

**Fig. S9** Local quality estimate of the built model of CDW-xyl-8.

**Table S1.** The information of the dairy cows.

**Table S2.** Metagenomic data assembly results.

**Table S3.** Open reading frame (ORF) data of each sample.

**Table S4.** The α-diversity values of microbiota in different dairy cow fecal sample groups.

**Table S5.** The xylanase genes number, GH family classification, gene sequences ID in our dataset, and Genbank accession numbers for the 34 candidate xylanase genes.

**Table S6.** Homology analysis of 34 predicted xylanase protein sequences by BLAST in NCBI.


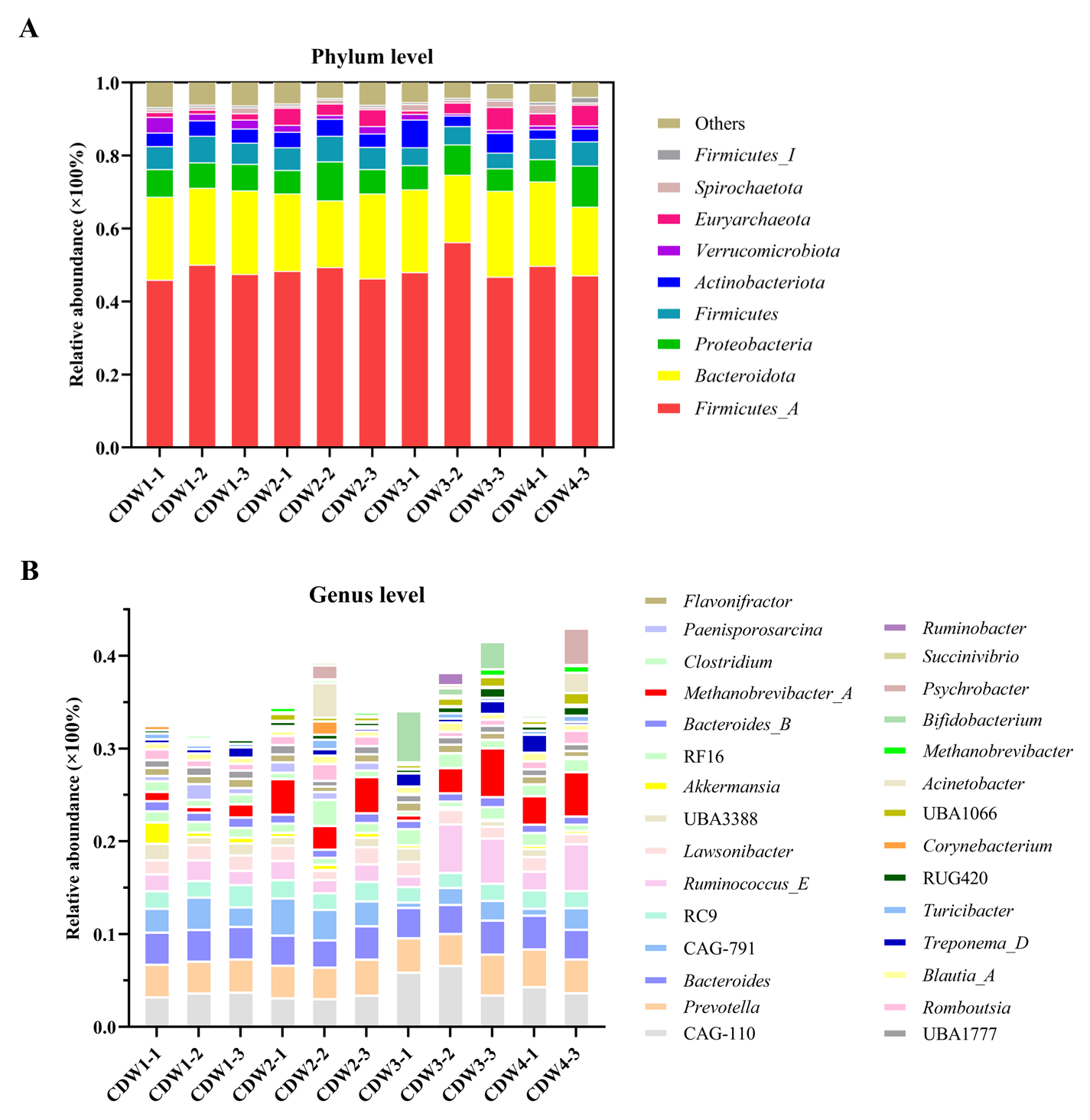


**Fig. S1 The** microbial compositions in different dairy cow fecal samples. Species diversity distribution of microbial communities at phylum (A) and genus (B) level in different cow fecal samples.


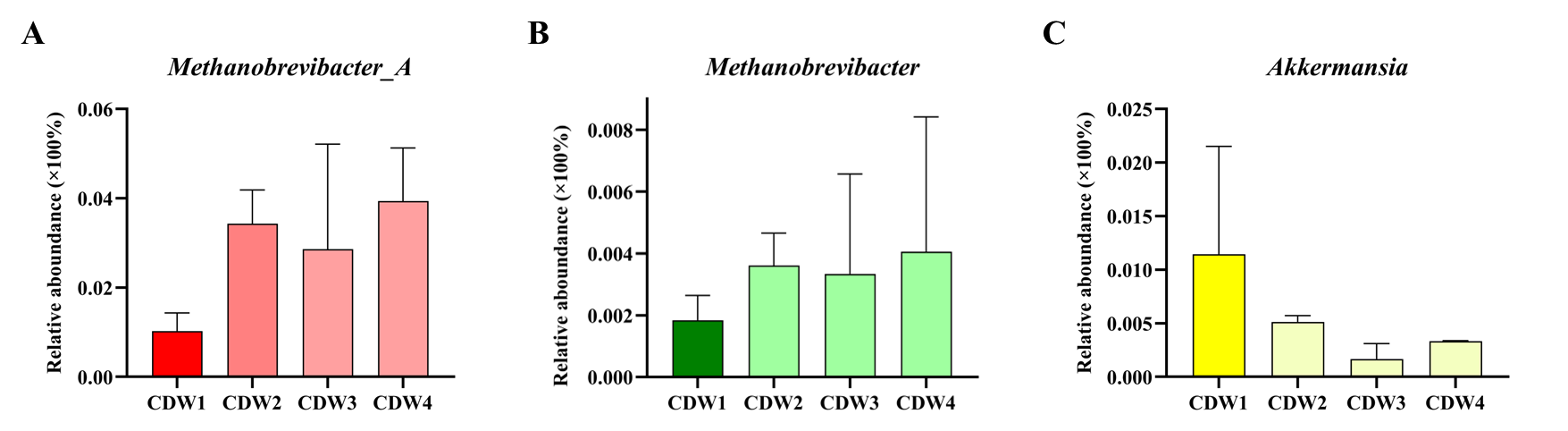


**Fig. S2** Three microbial genera of CDW-1 group were different from other groups.


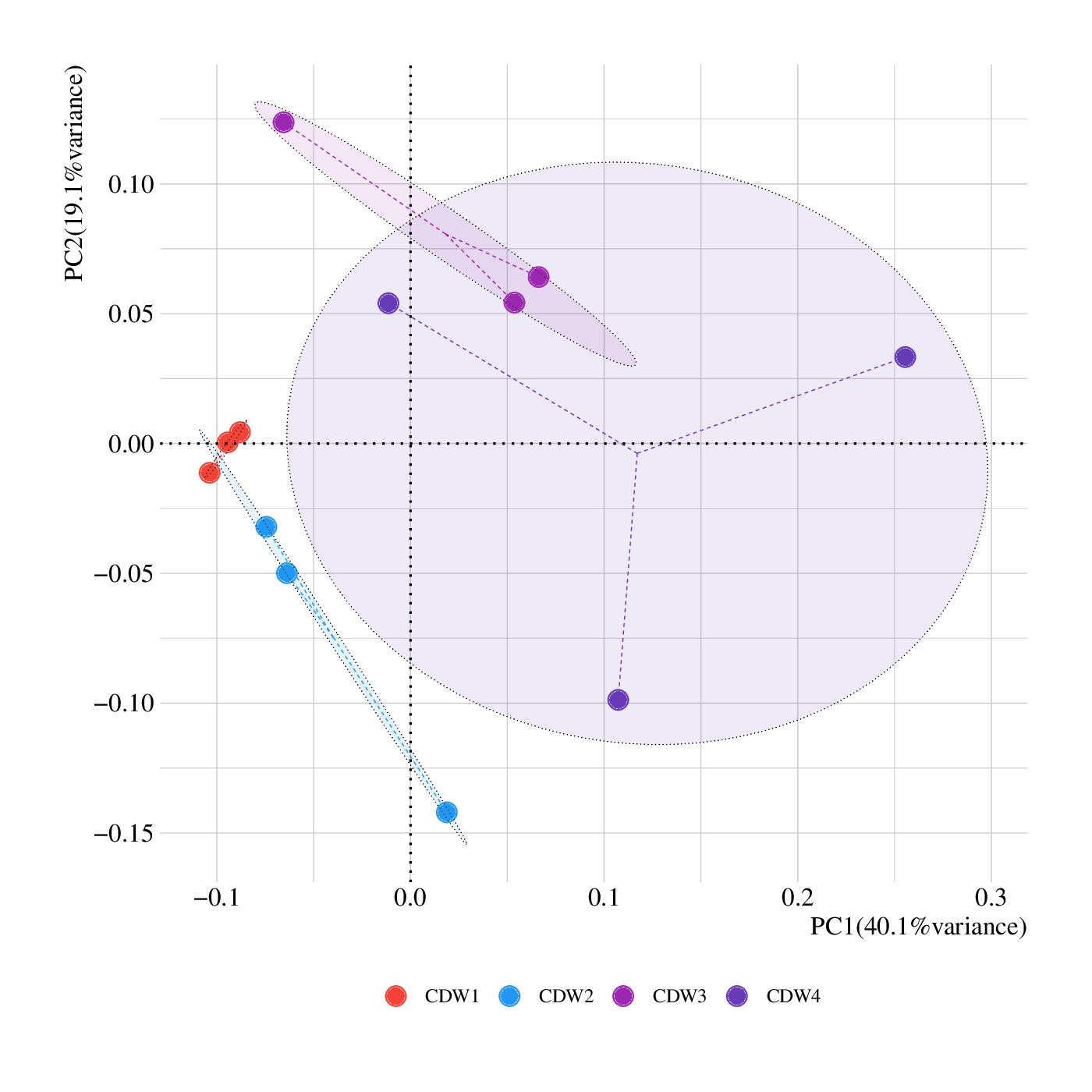


**Fig. S3** The β-diversity of microbiota in different dairy cow fecal samples. Principal coordinate analysis (PCoA) was used to analyze the differences in microbial composition between different samples.


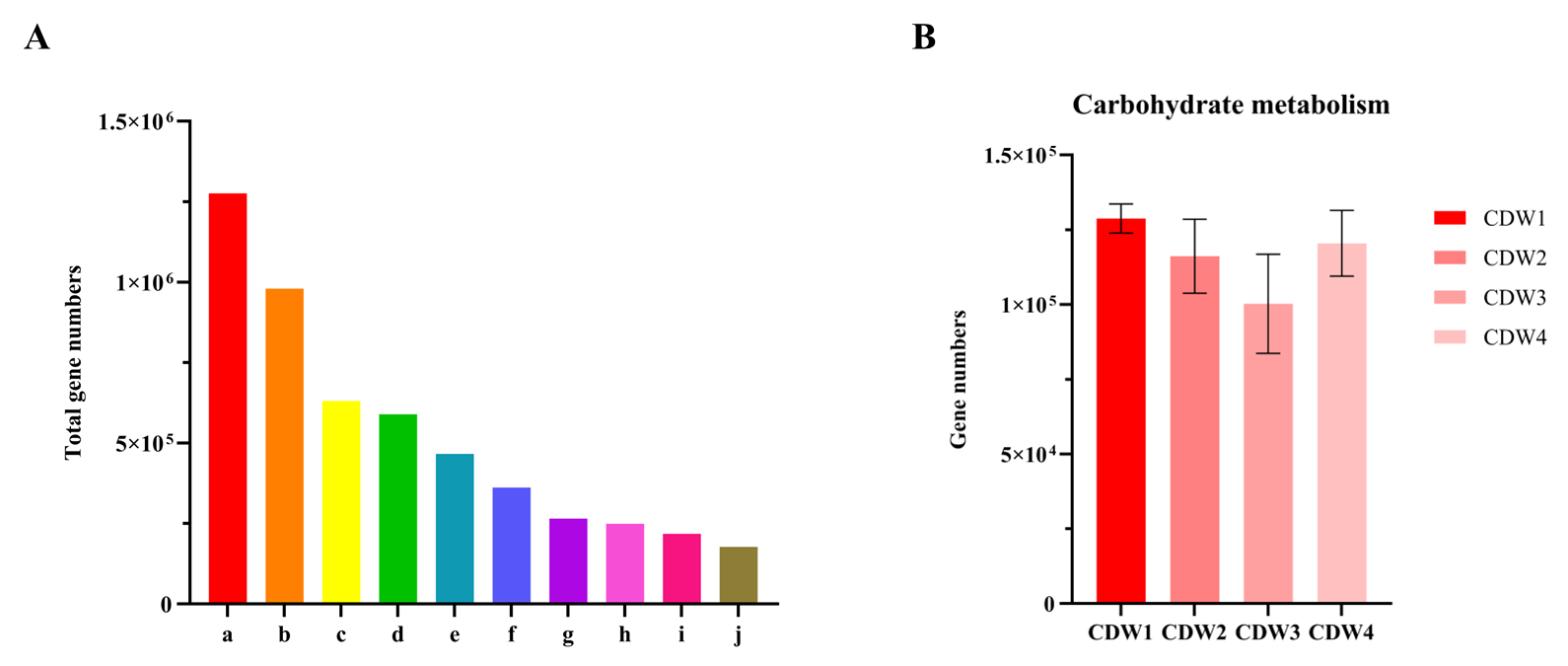


**Fig. S4** The annotated gene functions of KEGG pathway. (A) The total gene numbers of TOP10 metabolic pathways in dairy cow fecal samples. a: Carbohydrate metabolism; b: Amino acid metabolism; c: Energy metabolism; d: Metabolism of cofactors and vitamins; e: Nucleotide metabolism; f: Glycan biosynthesis and metabolism; g: Lipid metabolism; h: Metabolism of other amino acids; i: Biosynthesis of other secondary metabolites; j: Metabolism of terpenoids and polyketides. (B) The gene numbers of carbohydrate metabolic pathway of different sample groups.


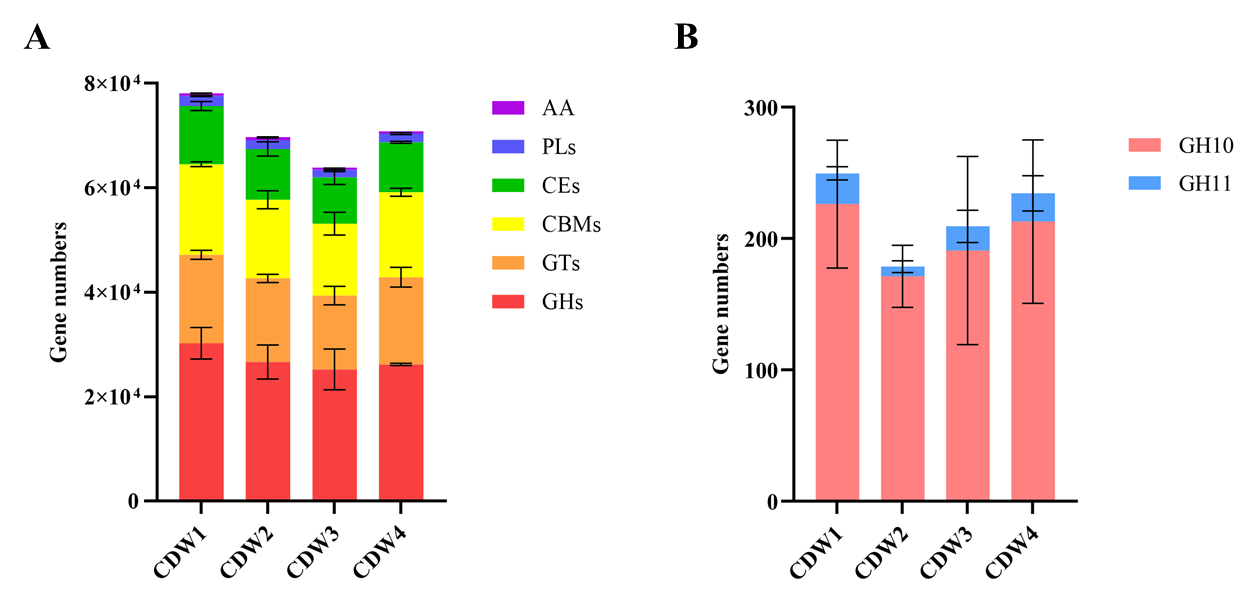


**Fig. S5** The numbers and classification of carbohydrate-active enzyme genes in different sample groups. (A) The numbers and classification of carbohydrate-active enzyme genes in four sample groups. GHs: Glycoside Hydrolases; GTs: Glycosyl Transferases; CBMs: Carbohydrate-Binding Modules; CEs: Carbohydrate Esterases; PLs: Polysaccharides Lyases; AA: Auxiliary Activities. (B) GH10 and GH11 family enzyme gene numbers in four groups of samples.


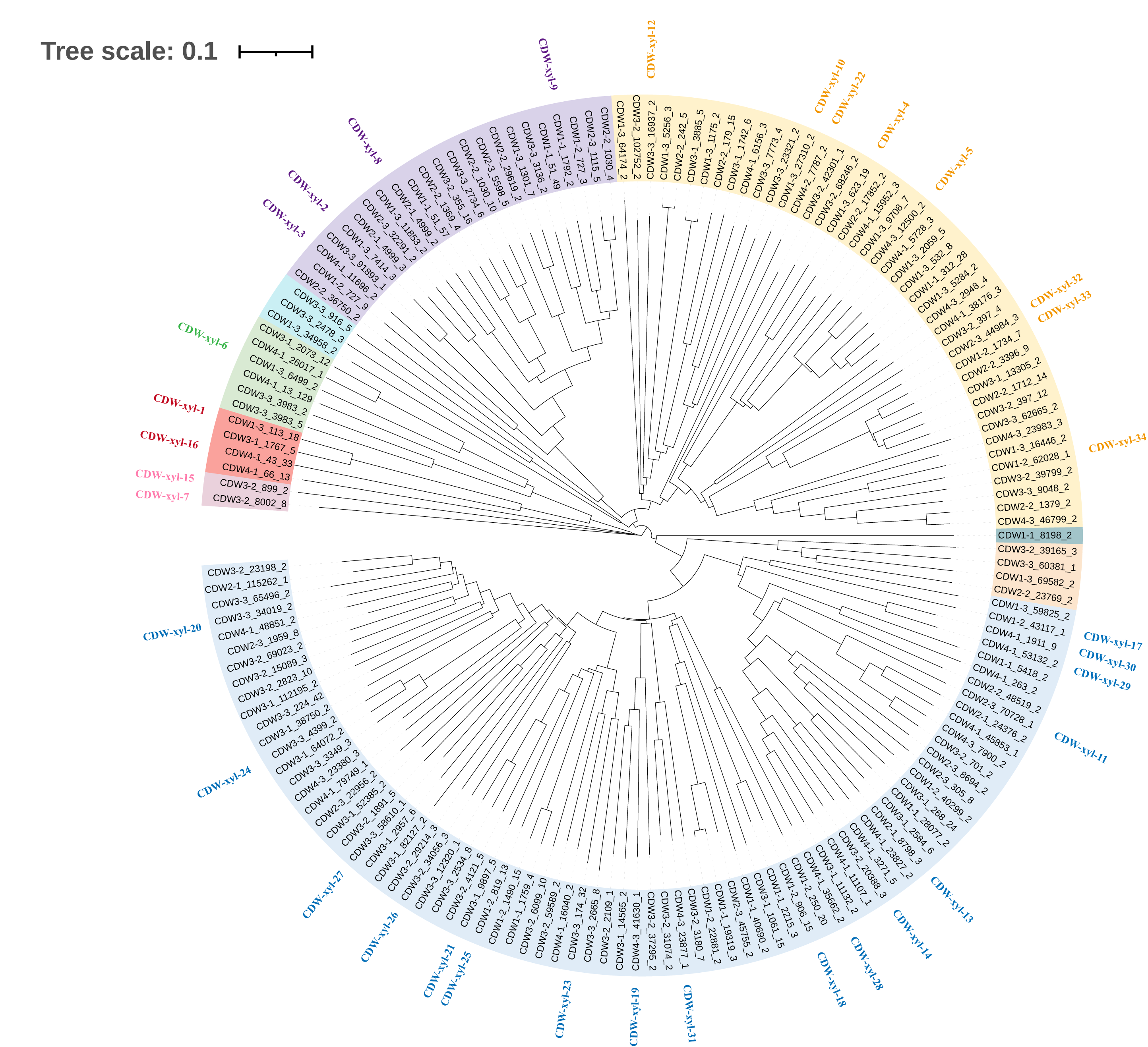


**Fig. S6** The phylogenetic tree of the predicted 163 genes (158 GH10 genes and 5 GH11 genes). 5 GH11 family genes are displayed in red and pink, GH10 family genes are shown in different colors according to their clustering, except CDW-xyl-7 (CDW3-2_8002_8) appear in pink. The 34 selected candidate xylanase genes have been marked in this figure corresponding to their gene numbers of the 163 genes.


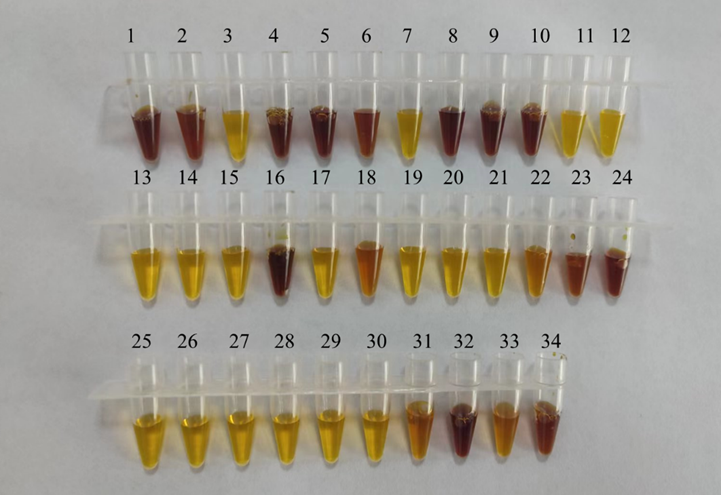


**Fig. S7** The crude enzyme activities of the selected 34 candidate xylanase genes.


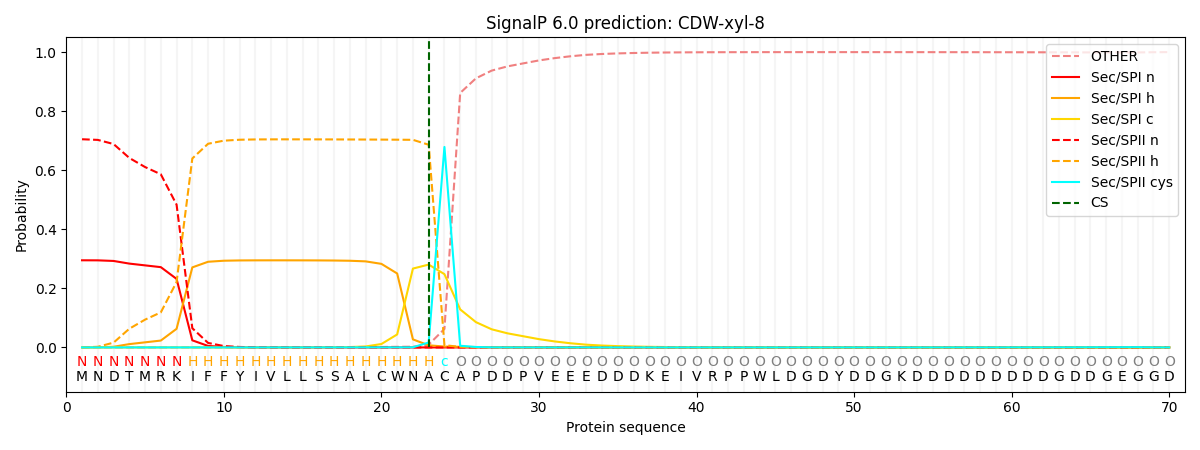


**Fig. S8** Signal peptide prediction of CDW-xyl-8 by SignalP 6.0 server.


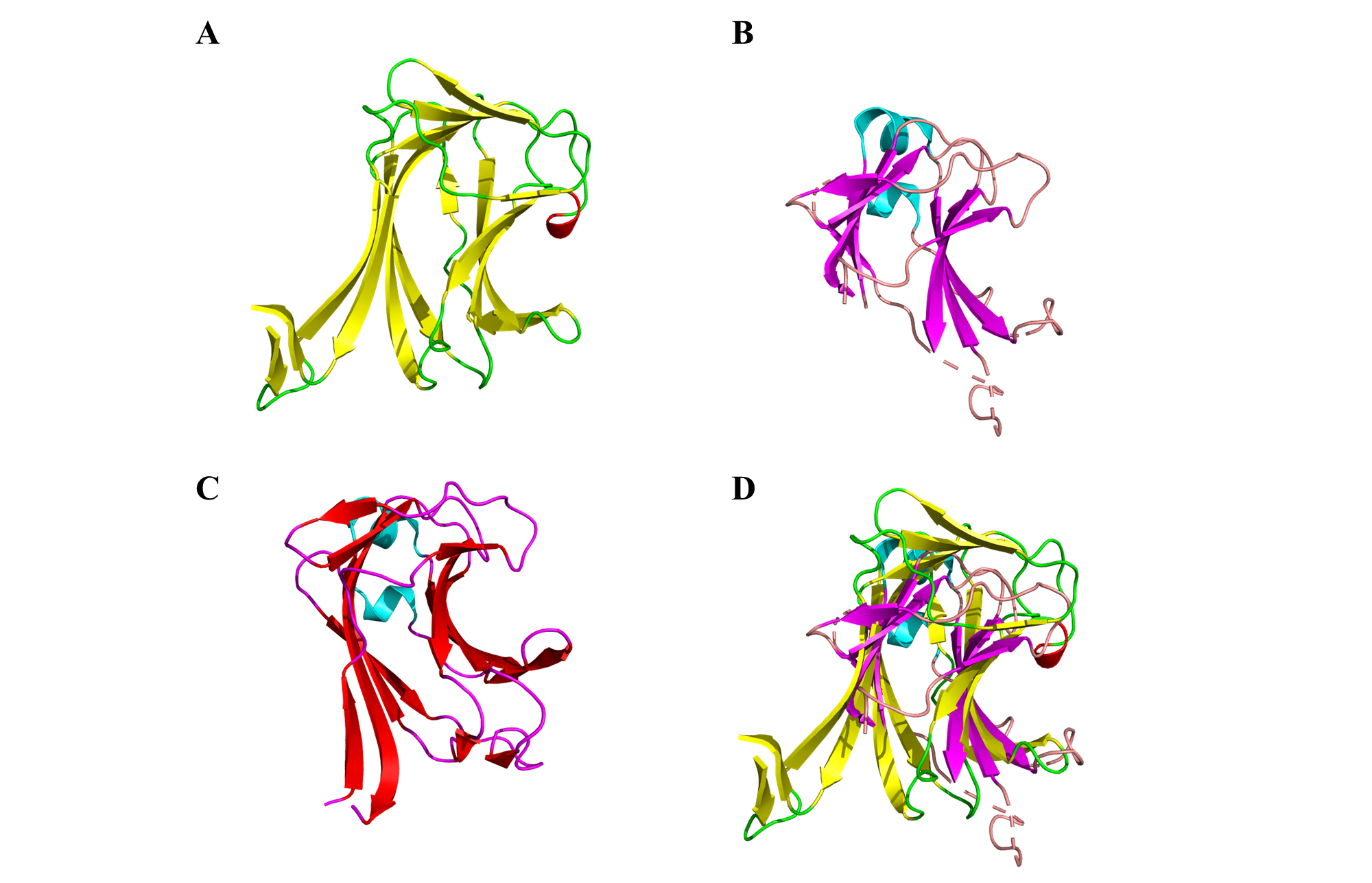


Fig. S9 Structure comparisons of the extra domain of CDW-xyl-8, the CBM15 module of C*j*Xyn10C, and a known structure of a CBM15 module of a xylanase Xyn10C. (A) The predicted cartoon structure of the extra domain of CDW-xyl-8 by AlphaFold2. (B) The CBM15 structure of C*j*Xyn10C (PDB ID: 1US3), a known GH10 family xylanase. (C) The cartoon structure of a CBM15 module of xylanase Xyn10C derived from *Pseudomonas cellulose*. (D) The superimposition of the predicted CDW-xyl-8 extra domain and the CBM15 module of C*j*Xyn10C.

**Table S1.** The information of the dairy cows.

| **Sample groups** | **Samples number** | **Species** | **Feed combinations** | **Dairy cow farm location** | **Dairy cow state** |
| --- | --- | --- | --- | --- | --- |
| CDW1 | CDW1-1  CDW1-2  CDW1-3 | Simmental | Animal feed concentrates, complete corn silage, wheat straw, and fermented herbal mixture (*Lactobacillus plantarum*, *Enterococcus faecium*, *Astragalus membranaceus*, *Poria cocus* Wolf, *Lonicera japonica* Thunb, *Crataegus pinnatifida* Bge.) | Guopo village, Taigong town, Weihui city, Henan | Healthy |
| CDW2 | CDW2-1  CDW2-2  CDW2-3 | Simmental | Corn, wheat bran, and corn hay | Guopo village, Taigong town, Weihui city, Henan | Healthy |
| CDW3 | CDW3-1  CDW3-2  CDW3-1 | Simmental | Animal feed concentrates, complete corn silage, and wheat straw | Tangzhuang village, Tangzhuang town, Weihui city, Henan | Healthy |
| CDW4 | CDW4-1  CDW4-2  CDW4-3 | Simmental | Animal feed concentrates, complete corn silage, and wheat straw | Shanbiao village, Tangzhuang town, Weihui City, Henan | Healthy |

Fermented herbal mixture consists of *Lactobacillus plantarum*, *Enterococcus faecium*, *Astragalus membranaceus*, *Poria cocus* Wolf, *Lonicera japonica* Thunb, *Crataegus* *pinnatifida* Bge.

**Table S2.** Metagenomic data assembly results.

| **Assembly** | **CDW1-1** | **CDW1-2** | **CDW1-3** | **CDW2-1** | **CDW2-2** | **CDW2-3** | **CDW3-1** | **CDW3-2** | **CDW3-3** | **CDW4-1** | **CDW4-2** | **CDW4-3** |
| --- | --- | --- | --- | --- | --- | --- | --- | --- | --- | --- | --- | --- |
| Contigs (>= 1000 bp) | 175256 | 162603 | 232970 | 185448 | 214090 | 212656 | 209894 | 197788 | 179783 | 181210 | 31808 | 177075 |
| contigs (>= 5000 bp) | 7594 | 7455 | 11543 | 7639 | 11904 | 9059 | 10374 | 14335 | 9209 | 6721 | 652 | 6362 |
| contigs (>= 10000 bp) | 2251 | 2450 | 3560 | 2002 | 4082 | 2338 | 3176 | 5215 | 2734 | 1809 | 132 | 1611 |
| contigs (>= 25000 bp) | 493 | 562 | 854 | 397 | 1094 | 491 | 647 | 1307 | 595 | 383 | 18 | 287 |
| contigs (>= 50000 bp) | 152 | 174 | 290 | 114 | 311 | 126 | 193 | 355 | 169 | 96 | 0 | 76 |
| Total length (>= 0 bp) | 1.8 Gb | 1.9 Gb | 2 Gb | 1.7 Gb | 1.6 Gb | 1.9 Gb | 1.8 Gb | 1.3 Gb | 1.5 Gb | 1.7 Gb | 0.5 Gb | 1.9 Gb |
| Total length (>= 1000 bp) | 0.4 Gb | 0.4 Gb | 0.5 Gb | 0.4 Gb | 0.5 Gb | 0.4 Gb | 0.5 Gb | 0.5 Gb | 0.4 Gb | 0.4 Gb | 0.1 Gb | 0.4 Gb |
| Total length (>= 5000 bp) | 87.5 Mb | 89.7 Mb | 140.2 Mb | 82.2 Mb | 151.4 Mb | 98.0 Mb | 118.5 Mb | 182.4 Mb | 104.8 Mb | 72.9 Mb | 5.6 Mb | 64.5 Mb |
| Total length (>= 10000 bp) | 51.5 Mb | 55.9 Mb | 86.6 Mb | 44.7 Mb | 98.6 Mb | 53.0 Mb | 70.0 Mb | 120.6 Mb | 61.6 Mb | 40.4 Mb | 0.2 Mb | 32.8 Mb |
| Total length (>= 25000 bp) | 25.6 Mb | 27.9 Mb | 47.2 Mb | 21.6 Mb | 54.4 Mb | 26.7 Mb | 33.3 Mb | 62.2 Mb | 30.3 Mb | 20.0 Mb | 0.6 Mb | 14.0 Mb |
| Total length (>= 50000 bp) | 13.8 Mb | 14.7 Mb | 27.9 Mb | 11.8 Mb | 27.6 Mb | 14.1 Mb | 17.9 Mb | 29.9 Mb | 15.7 Mb | 10.4 Mb | 0 | 6.6 Mb |
| contigs | 756796 | 757614 | 933017 | 784063 | 760528 | 890128 | 843437 | 617994 | 686898 | 784905 | 165484 | 801901 |
| Largest contig | 517039 | 266620 | 830690 | 493403 | 484848 | 515007 | 591049 | 406036 | 600716 | 657465 | 46473 | 234943 |
| Total length | 0.7 Gb | 0.7 Gb | 1.0 Gb | 0.8 Gb | 0.9 Gb | 0.9 Gb | 0.9 Gb | 0.8 Gb | 0.7 Gb | 0.8 Gb | 0.2 Gb | 0.8 Gb |
| GC (%) | 45.59 | 45.34 | 45.32 | 44.85 | 43.78 | 44.82 | 44.78 | 45.03 | 45.03 | 43.93 | 44.35 | 44.58 |
| N50 | 971 | 936 | 1054 | 977 | 1203 | 987 | 1035 | 1462 | 1087 | 949 | 823 | 916 |

**Table S3.** Open reading frame (ORF) data of each sample.

| **Sample**  **names** | **Sequence number** | **Sequence bases (bp)** | **Minimal length** | **Maximum length** | **Average length** |
| --- | --- | --- | --- | --- | --- |
| CDW1-1 | 5020498 | 1633897851 | 60 | 31272 | 325.45 |
| CDW1-2 | 5199424 | 1677753219 | 60 | 24345 | 322.68 |
| CDW1-3 | 4984724 | 1751453211 | 60 | 23766 | 351.36 |
| CDW2-1 | 4527441 | 1517636097 | 60 | 20739 | 335.21 |
| CDW2-2 | 3886148 | 1417195416 | 60 | 23292 | 364.68 |
| CDW2-3 | 4971048 | 1695612813 | 60 | 20943 | 341.1 |
| CDW3-1 | 4527723 | 1583705082 | 60 | 18420 | 349.78 |
| CDW3-2 | 2997355 | 1173835947 | 60 | 20808 | 391.62 |
| CDW3-3 | 3813525 | 1321270194 | 60 | 30273 | 346.47 |
| CDW4-1 | 4578354 | 1511469792 | 60 | 16524 | 330.13 |
| CDW4-2 | 1424459 | 422815335 | 60 | 10611 | 296.83 |
| CDW4-3 | 5312317 | 1697779263 | 60 | 25425 | 319.59 |

**Table S4.** The α-diversity values of microbiota in different dairy cow fecal sample groups.

| **Sample groups** | **Chao1** | **Shannon** | **Simpson** |
| --- | --- | --- | --- |
| CDW1 | 19729±1015 | 11.9±0.115 | 0.00149±0.00031 |
| CDW2 | 18850±877 | 11.7±0.265 | 0.00153±0.00061 |
| CDW3 | 17146±921 | 11.3±0.153 | 0.00316±0.00098 |
| CDW4 | 13363±8053 | 11±0.9 | 0.00291±0.00182 |

**Table S5.** The xylanase genes number, GH family classification, gene sequences ID in our dataset and Genbank accession numbers for the 34 candidate xylanase genes.

| Candidate xylanase genes number | GH family | Gene sequences ID | Genbank accession numbers |
| --- | --- | --- | --- |
| CDW-xyl-1 | GH11 | CDW1-3_113_18 | OR237237 |
| CDW-xyl-2 | GH10 | CDW1-3_7414_3 | OR237240 |
| CDW-xyl-3 | GH10 | CDW4-1_11696_2 | OR237242 |
| CDW-xyl-4 | GH10 | CDW1-3_623_19 | OR237245 |
| CDW-xyl-5 | GH10 | CDW4-3_12500_2 | OR237248 |
| CDW-xyl-6 | GH10 | CDW1-3_6499_2 | OR237262 |
| CDW-xyl-7 | GH10 | CDW3-2_8002_8 | OR237264 |
| CDW-xyl-8 | GH10 | CDW2-1_4999_2 | OR237268 |
| CDW-xyl-9 | GH10 | CDW1-1_1792_2 | OR237279 |
| CDW-xyl-10 | GH10 | CDW1-3_27310_2 | OR237281 |
| CDW-xyl-11 | GH10 | CDW2-3_70728_1 | OR237297 |
| CDW-xyl-12 | GH10 | CDW3-3_16937_2 | OR237301 |
| CDW-xyl-13 | GH10 | CDW2-1_8798_3 | OR237305 |
| CDW-xyl-14 | GH10 | CDW3-2_20388_3 | OR237306 |
| CDW-xyl-15 | GH11 | CDW3-2_899_2 | OR237310 |
| CDW-xyl-16 | GH11 | CDW4-1_43_33 | OR237311 |
| CDW-xyl-17 | GH10 | CDW1-2_43117_1 | OR237312 |
| CDW-xyl-18 | GH10 | CDW1-2_906_15 | OR237324 |
| CDW-xyl-19 | GH10 | CDW4-3_41630_1 | OR237325 |
| CDW-xyl-20 | GH10 | CDW3-3_34019_2 | OR237327 |
| CDW-xyl-21 | GH10 | CDW3-1_9897_5 | OR237333 |
| CDW-xyl-22 | GH10 | CDW4-2_7787_2 | OR237335 |
| CDW-xyl-23 | GH10 | CDW3-3_174_32 | OR237340 |
| CDW-xyl-24 | GH10 | CDW3-3_4399_2 | OR237341 |
| CDW-xyl-25 | GH10 | CDW1-2_819_13 | OR237348 |
| CDW-xyl-26 | GH10 | CDW3-2_34056_3 | OR237349 |
| CDW-xyl-27 | GH10 | CDW3-3_58610_1 | OR237352 |
| CDW-xyl-28 | GH10 | CDW4-1_35662_2 | OR237361 |
| CDW-xyl-29 | GH10 | CDW4-1_53132_2 | OR237363 |
| CDW-xyl-30 | GH10 | CDW4-1_1911_9 | OR237364 |
| CDW-xyl-31 | GH10 | CDW4-3_23877_1 | OR237367 |
| CDW-xyl-32 | GH10 | CDW2-3_44984_3 | OR237379 |
| CDW-xyl-33 | GH10 | CDW1-2_1734_7 | OR237383 |
| CDW-xyl-34 | GH10 | CDW1-2_62028_1 | OR237388 |

**Table S6.** Homology analysis of 34 predicted xylanase protein sequences by Blast in NCBI

| **34 candidate xylanase genes** | **Blast alignment results** | **The alignment sequence ID** | **Identity** |
| --- | --- | --- | --- |
| CDW-xyl-1 | Glycoside hydrolase family 11 protein [*Paludibacteraceae* bacterium] | MBR4970165.1 | 99.79% |
| CDW-xyl-2 | Endo-1,4-beta-xylanase [*Bacteroidaceae* bacterium] | MBO5951286.1 | 98.97% |
| CDW-xyl-3 | Endo-1,4-beta-xylanase [*Bacteroidales* bacterium] | MBR5211889.1 | 99.87% |
| CDW-xyl-4 | Endo-1,4-beta-xylanase [*Bacteroidaceae* bacterium] | MBO7266277.1 | 96.64% |
| CDW-xyl-5 | Endo-1,4-beta-xylanase [*Bacteroidaceae* bacterium] | MBQ8242623.1 | 91.57% |
| CDW-xyl-6 | Endo-1,4-beta-xylanase [*Paludibacteraceae* bacterium] | MBO5828303.1 | 99.70% |
| CDW-xyl-7 | Endo-1,4-beta-xylanase [*Clostridia* bacterium] | MBQ7292897.1 | 55.08% |
| CDW-xyl-8 | Endo-1,4-beta-xylanase [*Bacteroidales* bacterium] | MBR5211896.1 | 66.61% |
| CDW-xyl-9 | Endo-1,4-beta-xylanase [*Bacteroidales* bacterium] | MBQ3608254.1 | 70.81% |
| CDW-xyl-10 | Endo-1,4-beta-xylanase [*Treponema* sp.] | MBO5116278.1 | 69.17% |
| CDW-xyl-11 | Endo-1,4-beta-xylanase [*Alistipes* sp.] | MBQ2394129.1 | 98.27% |
| CDW-xyl-12 | Endo-1,4-beta-xylanase [*Treponema* sp.] | MBO5826223.1 | 99.22% |
| CDW-xyl-13 | Endo-1,4-beta-xylanase [*Alistipes* sp.] | MBQ4531845.1 | 99.42% |
| CDW-xyl-14 | Endo-1,4-beta-xylanase [*Alistipes* sp.] | MBO5962120.1 | 97.08% |
| CDW-xyl-15 | PA14 domain protein [*Bacteroides* sp. 2_1_33B] | EEY83904.1 | 44.66% |
| CDW-xyl-16 | Glycoside hydrolase family 11 protein [*Fibrobacteraceae* bacterium] | MBQ5610210.1 | 98.74% |
| CDW-xyl-17 | Endo-1,4-beta-xylanase [*Kiritimatiellae* bacterium] | MBQ9739606.1 | 67.31% |
| CDW-xyl-18 | Endo-1,4-beta-xylanase [*Paenibacillus* sp. YN15] | WP_113023306.1 | 53.17% |
| CDW-xyl-19 | Endo-1,4-beta-xylanase [*Clostridia* bacterium] | MBQ6701635.1 | 80.54% |
| CDW-xyl-20 | Endo-1,4-beta-xylanase [*Clostridia* bacterium] | MBR2651099.1 | 70.69% |
| CDW-xyl-21 | Endo-1,4-beta-xylanase [*Clostridia* bacterium] | MBQ2272736.1 | 98.66% |
| CDW-xyl-22 | Endo-1,4-beta-xylanase [*Clostridia* bacterium] | MBO7196939.1 | 90.16% |
| CDW-xyl-23 | Endo-1,4-beta-xylanase [*Oscillospiraceae* bacterium] | MBQ3146159.1 | 88.57% |
| CDW-xyl-24 | Endo-1,4-beta-xylanase [*Clostridia* bacterium] | MBQ9692497.1 | 56.84% |
| CDW-xyl-25 | Endo-1,4-beta-xylanase [*Clostridia* bacterium] | MBO5279711.1 | 97.74% |
| CDW-xyl-26 | Endo-1,4-beta-xylanase [*Clostridia* bacterium] | MBQ6053507.1 | 79.95% |
| CDW-xyl-28 | Endo-1,4-beta-xylanase [*Clostridia* bacterium] | MBQ5602058.1 | 99.53% |
| CDW-xyl-29 | Endo-1,4-beta-xylanase [*Alistipes* sp.] | MBQ2394839.1 | 82.99% |
| CDW-xyl-30 | Endo-1,4-beta-xylanase [*Alistipes* sp.] | MBQ2394839.1 | 94.68% |
| CDW-xyl-31 | Endo-1,4-beta-xylanase [*Clostridia* bacterium] | MBQ3506184.1 | 95.82% |
| CDW-xyl-32 | Endo-1,4-beta-xylanase [*Bacteroidaceae* bacterium] | MBR4301967.1 | 98.17% |
| CDW-xyl-33 | Endo-1,4-beta-xylanase [*Bacteroidales* bacterium] | MBR5299331.1 | 91.86% |
| CDW-xyl-34 | Endo-1,4-beta-xylanase [*Bacteroidaceae* bacterium] | MBR5841275.1 | 95.88% |
